# One-year experiment on the physiological response of the Mediterranean crustose coralline alga, *Lithophyllum cabiochae*, to elevated *p*CO_2_ and temperature

**DOI:** 10.1002/ece3.475

**Published:** 2013-02-13

**Authors:** Sophie Martin, Stéphanie Cohu, Céline Vignot, Guillaume Zimmerman, Jean-Pierre Gattuso

**Affiliations:** 1 CNRS-INSU, Laboratoire d'Océanographie de Villefranche-sur-MerBP 28, 06234, Villefranche-sur-Mer Cedex, France; 2 Université Pierre et Marie Curie - Paris 6, Observatoire Océanologique de Villefranche06230, Villefranche-sur-Mer Cedex, France; 3 CNRS, Laboratoire Adaptation et Diversité en Milieu MarinStation Biologique de Roscoff, Place Georges Teissier, 29688, Roscoff Cedex, France; 4 Université Pierre et Marie Curie - Paris 6, Laboratoire Adaptation et Diversité en Milieu MarinStation Biologique de Roscoff, Place Georges Teissier, 29688, Roscoff Cedex, France

**Keywords:** Calcification, coralligenous habitat, coralline algae, global warming, irradiance, ocean acidification, pCO_2_, photosynthesis, respiration, temperature

## Abstract

The response of respiration, photosynthesis, and calcification to elevated pCO_2_ and temperature was investigated in isolation and in combination in the Mediterranean crustose coralline alga *Lithophyllum cabiochae*. Algae were maintained in aquaria during 1 year at near-ambient conditions of irradiance, at ambient or elevated temperature (+3°C), and at ambient (*ca*. 400 μatm) or elevated pCO_2_ (*ca*. 700 μatm). Respiration, photosynthesis, and net calcification showed a strong seasonal pattern following the seasonal variations of temperature and irradiance, with higher rates in summer than in winter. Respiration was unaffected by pCO_2_ but showed a general trend of increase at elevated temperature at all seasons, except in summer under elevated pCO_2_. Conversely, photosynthesis was strongly affected by pCO_2_ with a decline under elevated pCO_2_ in summer, autumn, and winter. In particular, photosynthetic efficiency was reduced under elevated pCO_2_. Net calcification showed different responses depending on the season. In summer, net calcification increased with rising temperature under ambient pCO_2_ but decreased with rising temperature under elevated pCO_2_. Surprisingly, the highest rates in summer were found under elevated pCO_2_ and ambient temperature. In autumn, winter, and spring, net calcification exhibited a positive or no response at elevated temperature but was unaffected by pCO_2_. The rate of calcification of *L. cabiochae* was thus maintained or even enhanced under increased pCO_2_. However, there is likely a trade-off with other physiological processes. For example, photosynthesis declines in response to increased pCO_2_ under ambient irradiance. The present study reports only on the physiological response of healthy specimens to ocean warming and acidification, however, these environmental changes may affect the vulnerability of coralline algae to other stresses such as pathogens and necroses that can cause major dissolution, which would have critical consequence for the sustainability of coralligenous habitats and the budgets of carbon and calcium carbonate in coastal Mediterranean ecosystems.

## Introduction

Ocean acidification and climate change are currently under high scrutiny due to the threat that they represent for the biodiversity and function of marine ecosystems. Current increases in atmospheric carbon dioxide (CO_2_) and temperature proceed at unprecedented rates in the recent history of the Earth. Atmospheric CO_2_ concentration has risen from 280 ppm prior to the beginning of the industrial revolution to a current value of 388 ppm due to human activities and is expected to reach more than 700 ppm by the end of this century considering the Intergovernmental Panel on Climate Change (IPCC) scenarios (Solomon et al. 2007). Global average temperature at Earth's surface has risen by 0.7°C during the last century and is expected to rise by 3°C by 2100 (Solomon et al. 2007). Similar trends are expected for surface ocean CO_2_ partial pressure (pCO_2_) and temperature due to the oceanic uptake of anthropogenic CO_2_ (Sabine et al., 2004) and to the warming of the surface mixed layer (Levitus et al. 2005).

Increasing pCO_2_ in the surface ocean is likely to decrease pH by 0.2–0.4 units over the course of this century (Caldeira and Wickett 2005) which will cause major shifts in seawater chemistry, with an increase in the concentration of bicarbonate ions (HCO_3_^−^) and a decrease in the concentration of carbonate ions (CO_3_^2−^) and the saturation state of calcium carbonate (CaCO_3_). Such shifts are likely to affect both calcifying and photosynthetic marine organisms due to potential changes in their physiological processes of calcification and photosynthesis that both use dissolved inorganic carbon (*C*_T_: HCO_3_^−^, CO_3_^2−^ and CO_2_) as substrate. Although the physiological response of marine organisms to ocean acidification is variable among taxa and species (Doney et al. 2009; Ries et al. 2009), the decrease in the availability of CO_3_^2−^ is known to affect the ability of marine calcifiers to form their carbonate skeleton or shells by a decline in calcification rates. A recent meta-analysis demonstrated that calcification is generally negatively affected by ocean acidification (Kroeker et al. 2010). Seawater acidification is also likely to affect photosynthesis due to the shift in the relative proportions of CO_2_ and HCO_3_^−^, the two forms of *C*_T_ that can be used for photosynthesis. Algae can use dissolved CO_2_ entering the cell by diffusion as the carbon source for photosynthesis but most of them have carbon concentrating mechanisms (CCMs) which actively take up HCO_3_^−^ which is converted to CO_2_ in the cells. This is a powerful mechanism counteracting the limited availability of CO_2_ in seawater (Raven and Geider 2003). An increase in seawater pCO_2_ could thus enhance photosynthesis in plants that rely exclusively on CO_2_ diffusion (Kübler et al. 1999), while it would be less favorable to algae that use CCMs (Giordano et al. 2005).

The calcareous red coralline algae coralline algae (Corallinales, Rhodophyta) are of particular interest to investigate as they conduct both photosynthesis and calcification. They are also considered among the most sensitive calcifying organisms to respond to ocean acidification due to the high solubility for their high magnesian calcite skeleton. Coralline algae are absent in naturally acidified seawater where other calcifiers can survive (Hall-Spencer et al. 2008; Martin et al. 2008). Their recruitment (Agegian 1985; Kuffner et al. 2007) and growth (Agegian 1985; Jokiel et al. 2008; Hofmann et al. 2012) are both negatively affected under elevated pCO_2_. Most recent studies show that coralline calcification is negatively affected under elevated pCO_2_ (Anthony et al. 2008; Semesi et al. 2009; Gao and Zheng 2010; Büdenbender et al. 2011; Johnson and Carpenter 2012) and that this effect is exacerbated by further ocean warming (Anthony et al. 2008). However, some authors reported a significant pCO_2_ effect on calcification only in combination with increased temperature (Martin and Gattuso 2009) or a positive pCO_2_ effect under moderate levels with a parabolic response of calcification in response to increased pCO_2_ (Smith and Roth 1979; Ries et al. 2009). The response of photosynthesis and respiration to ocean acidification in coralline algae is poorly understood. To our knowledge, only one study has investigated the effect of increased pCO_2_ on respiration in coralline algae and showed no effect (Semesi et al. 2009). The response of coralline photosynthesis to ocean acidification is variable among species but most are negatively affected (Anthony et al. 2008; Gao and Zheng 2010; Hofmann et al. 2012) with a larger impact under elevated temperature (Anthony et al. 2008). Conversely, some authors found positive (Borowitzka 1981; Semesi et al. 2009) or parabolic (Borowitzka 1981) responses. Very few studies have investigated calcification, photosynthesis, and respiration all together in coralline algae. However, these processes are tightly linked and the formation of CaCO_3_ crystals in cell walls of coralline algae is suggested to be largely controlled by photosynthesis and respiration (Smith and Roth 1979; Borowitzka 1981; Gao et al. 1993; De Beer and Larkum 2001). Photosynthesis may stimulate calcification by providing an organic matrix in the cell walls where the nucleation of calcite crystals is thought to occur (Borowitzka 1981). Furthermore, photosynthesis and respiration are two processes that control pH and that may in turn influence calcification rates. Photosynthesis increases pH and thereby increases the CaCO_3_ saturation state, favoring calcification (Gao et al. 1993) while respiration decreases pH and act in the opposite direction by hindering calcification (De Beer and Larkum 2001). While most recent research has focused on the response of coralline algae to ocean acidification, their response to the combined rise in pCO_2_ and temperature has been poorly investigated. Marine organisms are adapted to live in specific environmental temperature ranges, and a rise in temperature is likely to have direct effects on their physiology. By making coralline algae more vulnerable to other stressors, elevated pCO_2_ could have a larger impact when combined with elevated temperature than alone (Anthony et al. 2008; Martin and Gattuso 2009; Diaz-Pulido et al. 2012).

Changes in calcification and primary production in coralline algae may have profound consequences for the ecosystems that they compose from polar regions to the tropics (Johansen 1981) and in which they are a major calcifying component of the marine benthos. Coralline algae are of particular ecological importance in shallow waters, inducing settlement and recruitment of numerous invertebrates and providing habitats for a high diversity of associated organisms (Johansen 1981). Their rigid structure contributes to the formation of numerous habitats such as rhodolith beds (Foster, 2001) or coralligenous habitats (Ballesteros 2006). Coralline algae are also of significant importance in the carbon and carbonate cycles of shallow coastal ecosystems, being major contributors to CO_2_ fluxes through high community photosynthesis and respiration (Martin et al. 2005, 2007) and through high CaCO_3_ production and dissolution (Barron et al. 2006; Martin et al. 2007).

A better understanding of how coralline algal photosynthesis, respiration, and calcification respond to ocean acidification and warming is critical to predicting how coralline algae-based community may change in response to global environmental changes. That response may also vary depending on changes in other environmental factors and in particular irradiance which is the third major physical variable that affects both photosynthesis and calcification. The response of coralline algae to ocean acidification and/or warming has mainly been investigated through short-term (a few days to a few weeks) experimental approach, therefore neglecting the potential for physiological acclimation. In this study, we investigate, through a long-term (1 year) experiment, the combined effects of elevated pCO_2_ and temperature on respiration, photosynthesis, and net calcification in the crustose coralline algae, *Lithophyllum cabiochae*, which is one of the main calcareous components of coralligenous communities in the Mediterranean Sea. We hypothesize that future changes in pCO_2_ and temperature will incur a physiological stress in *L. cabiochae* thereby affecting its metabolic rates. We report on the response to elevated pCO_2_ and temperature in the four seasons to assess how seasonal variations of temperature and irradiance may interact with global environmental changes.

## Material and Methods

### Biological material

**Figure 1:**
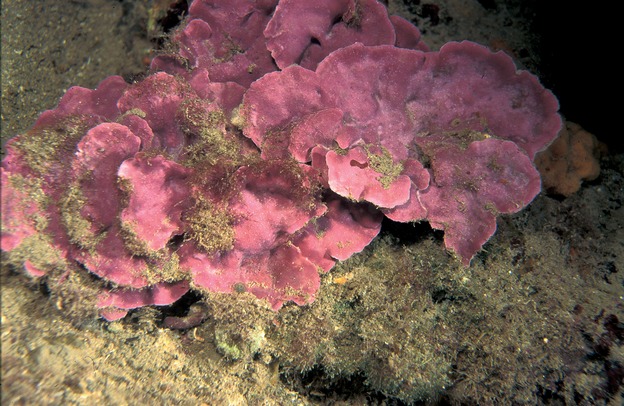
The crustose coralline alga *Lithophyllum cabiochae*. Photo by David Luquet.

Specimens of the crustose coralline alga, *Lithophyllum cabiochae* (Boudouresque & Verlaque) Athanasiadis (Fig. 1) were collected in the coralligenous community at *ca*. 25 m depth in the Bay of Villefranche (NW Mediterranean Sea, France; 43°40.73′N, 07°19.39′E) on 10 July 2006 and transported in thermostated tanks to the Villefranche Oceanography Laboratory. Algae were thoroughly cleaned of epiphytic organisms without causing any damage to the thalli. Flat thalli in the size range of 15–30 cm^2^ (*ca*. 0.35 g dry weight cm^−2^) were selected for the experiments. The algal surface area was determined from photographs using the software Image J Version 1.37v.

### Chlorophyll *a* analysis

Fragments of each thallus of less than 0.5 cm^2^ were taken at the end of each seasonal experiment for chlorophyll *a* (Chl *a*) analyses. Thallus fragments were photographed for surface determination and immediately frozen at −80°C pending analysis. Fragments were ground in 10 mL 90% acetone with cold mortar and pestle on an ice bath in the dark. The extract was poured into 15 mL centrifuge tubes and placed in the dark at 4°C overnight. After centrifugation at 4000 rpm for 20 min, total Chl *a* concentration in the supernatant was determined according to the method of Strickland and Parsons (1972) using a Turner Design 10-AU fluorometer. Two successive extractions were necessary for a complete Chl *a* extraction.

### Experimental setup

**Figure 2:**
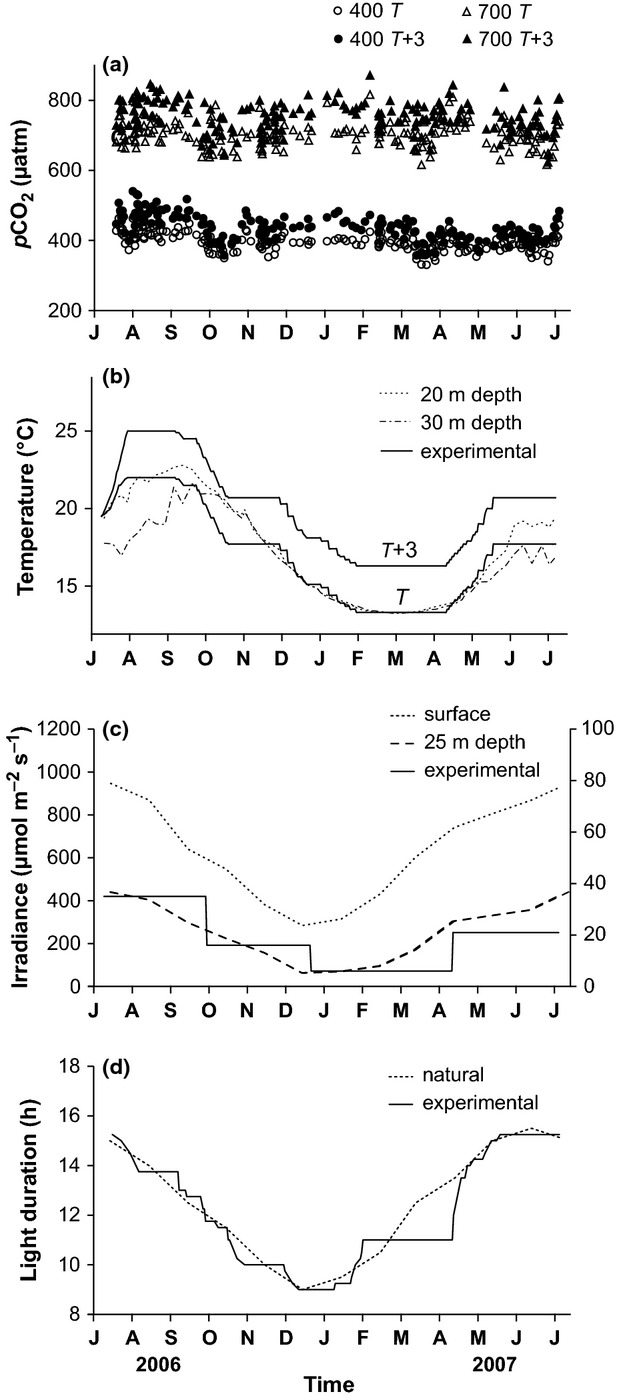
Changes in pCO_2_, temperature, irradiance, and photoperiod in the field from July 2006 to July 2007 and in the experimental tanks. *T*, ambient temperature; *T* + 3, elevated temperature (+3°C); irradiance at the surface, left axis; irradiance at 25 m depth and experimental irradiance, right axis.

Algae were randomly assigned in four 26-L aquaria (10–11 algae per aquarium) and grown during 1 year (July 2006–July 2007) in controlled conditions of pCO_2_ and temperature. A crossed (2 pCO_2_ × 2 temperature levels) experimental design was set up using four independent aquaria kept at ambient (*ca*. 400 μatm) or elevated pCO_2_ (*ca*. 700 μatm; Fig. 2a) and at ambient temperature (*T*, *i.e*. the in situ temperature that the algae experience at 25 m depth in the Bay of Villefranche) or elevated temperature (*T* + 3°C). There were therefore four treatments:

1. ambient pCO_2_ and ambient temperature (control, labeled 400 *T*),
2. ambient pCO_2_ and elevated temperature (400 *T +* 3),
3. elevated pCO_2_ and ambient temperature (700 *T*),
4. elevated pCO_2_ and elevated temperature (700 *T +* 3).

Aquaria were continuously supplied with Mediterranean seawater at a rate of 13 L h^−1^ from two 110-L header tanks in which pCO_2_ was adjusted by bubbling ambient air (ambient pCO_2_) or CO_2_-enriched air (elevated pCO_2_) obtained by mixing pure CO_2_ to ambient air. Temperature was gradually changed to the desired seasonal experimental value (*T* = 22.0°C in summer, 17.7°C in autumn, 13.3°C in winter, and 17.7°C in spring) and maintained constant 1 month prior to physiological measurement (Fig. 2b). Ambient temperature (*T*) corresponded to the temperature at 25 m depth in the Bay of Villefranche. It was regularly modified according to mean changes of temperature measured between 1995 and 2006 at 20 and 30 m depth by the Service d'Observation de la Rade de Villefranche, SO-Rade, of the Observatoire Océanologique and the Service d'Observation en Milieu Littoral, SOMLIT/CNRS-INSU (Fig. 2b). Temperature was controlled in each aquarium to within ±0.1°C using temperature controllers (Corema) connected to 150 W submersible heaters. Irradiance was set to the mean in situ daily irradiance at 25 m depth in the Bay of Villefranche. It was calculated from surface irradiance using attenuation coefficients measured during the experimental period. Surface irradiance (photosynthetically available radiations, PAR; in μmol photons m^−2^ s^−1^) was measured using a flat quantum sensor (LI-COR, LI-192SA) set on the top of the *Sémaphore* of Saint-Jean-Cap-Ferrat, located near the sampling station. The attenuation coefficients (*K*_PAR_, mean ± SD = 0.14 ± 0.2 m^−1^) were calculated according to Kirk (1983) from irradiance profiles carried out monthly from the RV *Sagitta* in the Bay of Villefranche using an underwater flat quantum sensor (LI-COR, LI-192SA). The experimental irradiance was adjusted seasonally to 35, 16, 6, and 21 μmol m^−2^ s^−1^ in summer, autumn, winter, and spring, respectively, using neutral density filters (Fig. 2c). The light source consisted of two 39 W fluorescent tubes (JBL Solar Ultra Marin Day) above each aquaria. The photoperiod was adjusted weekly to the desired L:D (Light:Dark) ratio according to natural fluctuations. It varied from 9:15 in December to 15:9 in June and was maintained constant at 14:10, 10:14, 11:13, and 15:9 during the summer, autumn, winter, and spring seasonal experiments, respectively (Fig. 2d). To avoid undesirable “tank” effects, each aquarium was carefully cleaned every week and each header tank was cleaned every 3 weeks to prevent the growth of epiphytes and fouling communities or the accumulation of detritus. This maintenance and the high seawater renewal (50% h^−1^) prevented any major change in seawater composition. For more details on the experimental set up and measurements of the carbonate chemistry, see Martin and Gattuso (2009). The mean seasonal parameters of the carbonate chemistry in each aquarium are given in Table 1.

**Table 1:** Parameters of the carbonate system in each treatment and season

				CO_2_	HCO_3_^−^	CO_3_^2−^	*C*_T_		
						
	pH_T_	*A*_T_ (μmol kg^−1^)	pCO_2_ (μatm)	(μmol kg^−1^)				Ω_c_	Ω_a_
400 *T*									
Summer	8.06 (0.00)	2538 (4)	424 (3)	12.9 (0.1)	1944 (3)	246 (1)	2203 (2)	5.75 (0.03)	3.76 (0.02)
Autumn	8.09 (0.00)	2526 (5)	388 (3)	13.0 (0.1)	1962 (5)	233 (2)	2208 (3)	5.43 (0.05)	3.53 (0.03)
Winter	8.10 (0.00)	2540 (2)	386 (3)	14.9 (0.1)	2044 (3)	203 (1)	2262 (2)	4.74 (0.02)	3.04 (0.02)
Spring	8.09 (0.00)	2483 (3)	384 (3)	13.3 (0.1)	1947 (4)	220 (2)	2180 (2)	5.16 (0.04)	3.34 (0.02)
400 *T* + 3									
Summer	8.01 (0.00)	2541 (4)	475 (4)	13.4 (0.1)	1940 (3)	249 (1)	2203 (2)	5.84 (0.03)	3.86 (0.02)
Autumn	8.06 (0.00)	2533 (6)	425 (4)	13.1 (0.2)	1949 (6)	241 (2)	2203 (3)	5.64 (0.06)	3.69 (0.04)
Winter	8.06 (0.00)	2540 (2)	426 (5)	15.0 (0.2)	2029 (4)	210 (1)	2254 (2)	4.91 (0.03)	3.17 (0.02)
Spring	8.06 (0.00)	2484 (4)	412 (4)	13.1 (0.1)	1925 (4)	230 (2)	2168 (2)	5.40 (0.04)	3.52 (0.02)
700 *T*									
Summer	7.87 (0.00)	2543 (2)	714 (4)	21.7 (0.1)	2124 (2)	174 (1)	2319 (1)	4.07 (0.02)	2.66 (0.01)
Autumn	7.88 (0.00)	2521 (4)	695 (6)	23.4 (0.2)	2144 (3)	155 (1)	2322 (2)	3.62 (0.03)	2.35 (0.02)
Winter	7.87 (0.00)	2539 (1)	709 (5)	27.3 (0.2)	2220 (2)	130 (1)	2378 (1)	3.04 (0.02)	1.95 (0.01)
Spring	7.87 (0.00)	2483 (4)	693 (6)	24.0 (0.3)	2126 (4)	146 (2)	2296 (3)	3.44 (0.04)	2.23 (0.03)
700 *T* + 3									
Summer	7.84 (0.00)	2546 (3)	779 (6)	22.0 (0.2)	2113 (2)	180 (1)	2315 (2)	4.22 (0.02)	2.79 (0.02)
Autumn	7.86 (0.00)	2530 (3)	733 (6)	22.7 (0.3)	2128 (4)	166 (2)	2317 (3)	3.89 (0.04)	2.54 (0.03)
Winter	7.85 (0.00)	2545 (2)	763 (5)	26.8 (0.2)	2208 (2)	139 (1)	2374 (1)	3.24 (0.02)	2.09 (0.01)
Spring	7.85 (0.00)	2487 (4)	738 (6)	23.4 (0.2)	2110 (4)	156 (1)	2289 (2)	3.66 (0.03)	2.38 (0.02)

The values reported are means (SE) of 8–17 data for total alkalinity (*A*_T_) and 40–55 data for pH_T_ (on the total scale) and the other parameters. Mean pH_T_ values are calculated by transformation of pH_T_ to [H^+^] and reconversion of mean [H^+^] to pH_T_. The CO_2_ partial pressure (*p*CO_2_), the concentrations of CO_2_, CO_3_^2−^, HCO_3_^−^, and dissolved inorganic carbon (*C*_T_), and the saturation state of seawater with respect to calcite (Ω_c_) and aragonite (Ω_a_) are calculated from pH_T_, temperature, salinity, and mean seasonal *A*_T_.

### Physiological measurements

Physiological measurements were performed in summer 2006 (23 August – 7 September 2006), autumn 2006 (14–29 November 2006), winter 2007 (1 March–3 April 2007), and spring 2007 (11 June–7 July 2007).

**Figure 3:**
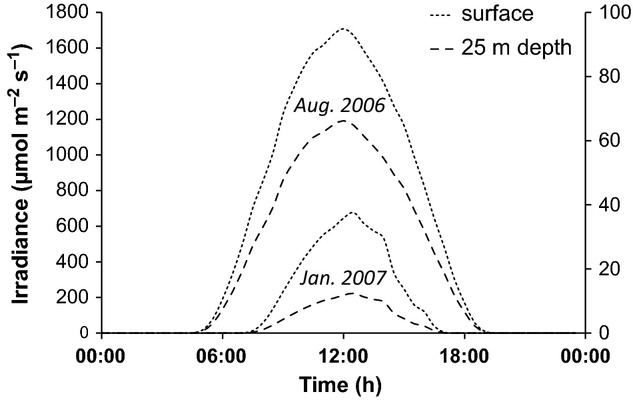
Evolution of daily mean irradiance at the surface (left axis) and at 25 m depth (right axis) in summer (August 2006) and winter (January 2007) in the Bay of Villefranche.

Only healthy (totally pink) algae were considered for the experiment, excluding those with necroses that appeared at the end of the summer period at elevated temperature (Martin and Gattuso 2009). Five algae were selected per treatment (except in the 700 *T +* 3 treatment in winter and spring where *n* = 4). When necroses occurred, algae were replaced by healthy specimens from the remaining pool of algae in the aquaria. Algae were incubated individually in closed Perspex chambers filled with *ca*. 200 mL of seawater from the aquaria and continuously stirred with a magnetic stirring bar. The chambers were placed inside the aquaria in order to control temperature. Clear chambers were used to assess net production (*P*_n_) and calcification (*G*) in the light while chambers with a dark plastic cover were used to assess dark respiration (*R*_d_) and calcification (*G*_d_). Incubations were conducted in the light at the seasonal ambient irradiance and in the dark. Additionally, in summer and winter, algae were incubated at different irradiance levels in the range of those at 25 m depth in the Bay of Villefranche (Fig. 3) calculated from surface irradiance and using attenuation coefficients of photosynthetically available radiations (*K*_PAR_ of 0.13 and 0.16 m^−1^ in summer and winter, respectively) as described in Martin and Gattuso (2009). The irradiance levels were adjusted using neutral density filters and controlled with a flat quantum sensor (LI-COR, LI-192SA, Li-COR Inc., Lincoln, USA). Incubations took place between 09:00 and 19:00 and were carried out for 1.5–3 h according to the season and the irradiance levels. The algae were acclimated at the desired irradiance level for at least 2 h prior to the incubation and were incubated only once a day. In the dark, algae were incubated after exposure to the ambient irradiance.

The concentration of dissolved oxygen (O_2_, μmol L^−1^) was continuously measured inside the chamber using Clark-type Strathkelvin 1302 oxygen electrodes connected to a Strathkelvin 782 oxygen meter. Water samples were taken at the beginning and at the end of the incubations for measurements of pH_T_ (pH on the total scale) and total alkalinity (A_T_) as described in Martin and Gattuso (2009). The concentration of dissolved inorganic carbon (*C*_T_) was determined from pH_T_, A_T_, temperature and salinity using the *R* package seacarb (Proye and Gattuso 2003).

*P*_n_ and *R*_d_ expressed in terms of O_2_ production and consumption, respectively (in μmol O_2_ cm^−2^ h^−1^) were calculated as:


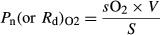


where *sO*_2_ is the slope of the linear regression line for change in O_2_ versus time (μmol L^−1^ h^−1^), *V* is the volume of the chamber (l) and *S* is the surface area of the thallus (cm^2^).

Gross production (*P*_g_) was calculated as:


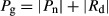


The changes in *C*_T_ during the incubations are controlled by the metabolism of organic (photosynthesis and respiration) and inorganic carbon (calcification and dissolution). The precipitation of 1 mol of CaCO_3_ decreases *C*_T_ by 1 mol and A_T_ by 2 eq according to:


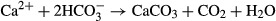


*G* and *G*_d_ (μmol CaCO_3_ cm^−2^ h^−1^) were calculated using the alkalinity anomaly technique (Smith and Key 1975) as:


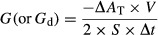


where Δ*A*_T_ is the difference between initial and final A_T_ values (μeq L^−1^) and Δ*t* is the incubation time (h).

*P*_n_ and *R*_d_ expressed in terms of CO_2_ fixation and release, respectively (in μmol C cm^−2^ h^−1^) were calculated as:


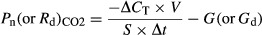


where Δ*C*_T_ is the difference between the initial and final *C*_T_ values (μmol L^−1^).

*P*_n_ and *G* measured at different irradiance (*E*) levels were fitted to the *P*_n_ (or *G*) versus *E* function of Platt et al. (1980) modified by the addition of a dark respiration (*R*_d_) or calcification (*G*_d_) term:


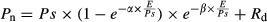



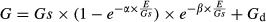


where *P*_s_ and *G*_s_ are scaling parameters defined as the maximum rates of photosynthesis (or calcification) in the absence of photoinhibition (or calcification inhibition under high irradiance), *α* is the initial slope of the light curve, and *β* is the photoinhibition coefficient.

The maximum rates of *P*_g_ (or gross calcification) at light saturation, *P*_g_^max^ (or *G*_g_^max^) are derived as (Harrison and Platt 1986):


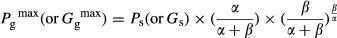


The maximum rate of *P*_n_ (or *G*), *P*_n_^max^ (or *G*^max^) are calculated as:


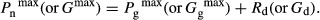


The saturating irradiance (*E*_k_, μmol photons m^−2^ s^−1^) is expressed as:


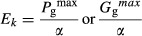


And the compensation irradiance (*E*_c_, μmol photons m^−2^ s^−1^), as:


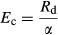


### Data analyses

The effect of pCO_2_ and temperature were assessed by two-way ANOVAs and followed by Tukey HSD post hoc tests or Tukey HSD post hoc tests for unequal sample sizes (Spjotvoll/Stoline test) to separate sets of homogeneous data. When necessary, data were log-transformed to meet ANOVA requirements of normal distribution (Shapiro-Wilks test) and equality of variance (Levene test). Independent ANOVAs were performed at each season as measurements were not repeated seasonally on the same algae (replacement in case of necroses). The probability levels were adjusted for repeated analyses using a Bonferroni correction (*α* set to 0.05 was divided by the number of analyses).

Results are expressed as mean ± standard error of the mean (SE).

## Results

### Respiration

**Figure 4:**
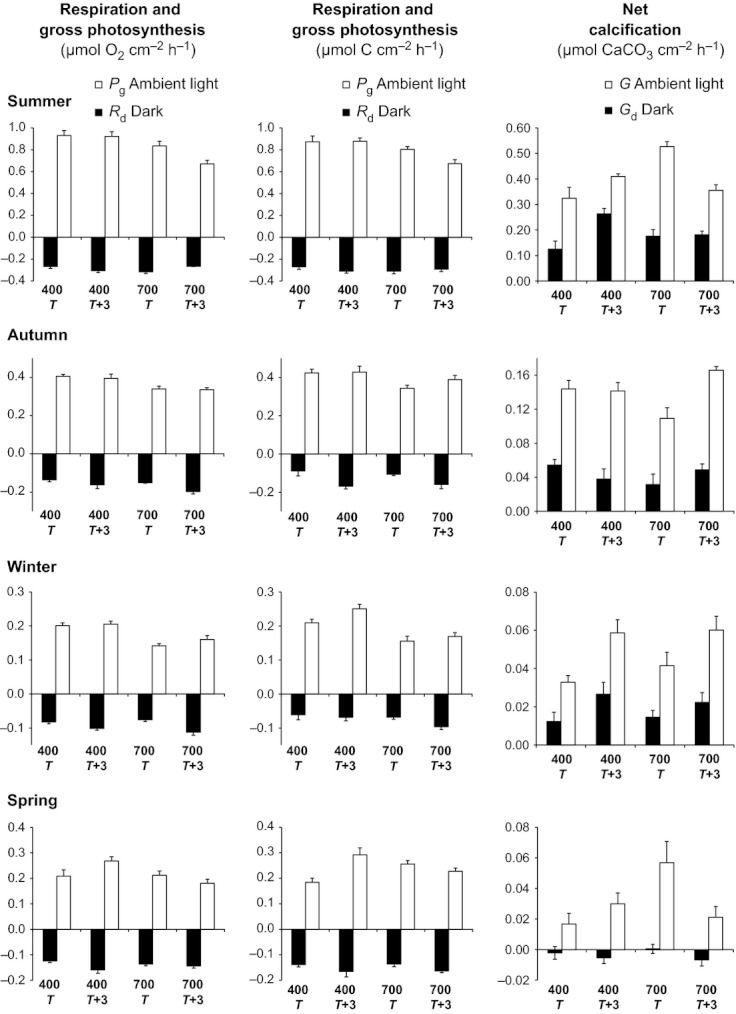
Gross production, respiration, and calcification rates of *Lithophyllum cabiochae* in the dark and at the experimental irradiance in the four treatments (400 *T*, 400 *T* + 3, 700 *T*, and 700 *T* + 3) in summer, autumn, winter, and spring. Gross production and respiration are expressed in terms of O_2_ release and CO_2_ fixation (negative values for respiration correspond to O_2_ consumption and CO_2_ release). Data are means ± SE (*n* = 5, except for the 700 *T* + 3 treatment in winter and spring, where *n* = 4).

Dark respiration (*R*_d_) presented a strong seasonal pattern following temperature variations. The highest rates were measured in summer (0.27–0.32 μmol cm^−2^ h^−1^ in terms of both O_2_ consumption and CO_2_ release), while the lowest rates (about threefold lower) were found in winter (0.06–0.11 μmol cm^−2^ h^−1^; Fig 4). Intermediate values were measured in autumn (0.09–0.20 μmol cm^−2^ h^−1^) and spring (0.14 to 0.17 μmol cm^−2^ h^−1^).

The general trend in *R*_d_ for the four seasons was an increase with the 3°C rise in temperature except in summer under 700 μatm where *R*_d_ declined at elevated temperature. Significant main effects of temperature were detected in both autumn and winter (Table 2). *R*_d_ was not affected by pCO_2_ whatever the season.

**Table 2:** Summary of two-way ANOVAs followed by Tukey HSD post hoc tests testing the effect of pCO_2_ and temperature on *Lithophyllum cabiochae* metabolism in the dark and at the culture irradiance levels and Chl *a* content at each season

	ANOVA data										
					
		Source of variation						Tukey HSD test			
			
		pCO_2_		temperature		pCO_2_ × temperature					
								
	df	F	*P*	F	*P*	F	*P*	400 *T*	400 *T*+3	700 *T*	700 *T*+3
**Summer**											
Dark											
*R*_d_ (O_2_)	(1,16)	0.030	0.864	0.085	0.775	6.864	0.019	nd			
*R*_d_ (CO_2_)	(1,16)	0.153	0.700	0.320	0.579	1.820	0.196	nd			
*G*_d_ (CaCO_3_)	(1,16)	0.432	0.520	8.749	**0.009**	6.913	0.018	a	b	ab	ab
Light											
*P*_n_ (O_2_)	(1,16)	33.977	**<0.001**	6.524	0.021	1.390	0.256	a	a	ab	b
*P*_n_ (CO_2_)	(1,16)	41.200	**<0.001**	10.160	**0.006**	2.610	0.126	a	a	ab	b
*P*_g_ (O_2_)	(1,16)	17.983	**<0.001**	4.243	0.056	3.853	0.067	a	a	ab	b
*P*_g_ (CO_2_)	(1,16)	13.216	**0.002**	2.747	0.117	3.304	0.088	a	a	ab	b
*G* (CaCO_3_)	(1,16)	8.346	**0.011**	2.743	0.117	24.690	**<0.001**	a	ab	b	a
Chl *a*	(1,16)	0.017	0.897	2.895	0.108	0.617	0.444	nd			
**Autumn**											
Dark											
*R*_d_ (O_2_)	(1,16)	3.552	0.078	7.136	0.017	0.595	0.452	nd			
*R*_d_ (CO_2_)	(1,16)	0.035	0.853	12.203	**0.003**	0.448	0.513	nd			
*G*_d_ (CaCO_3_)	(1,16)	0.369	0.552	0.000	0.984	2.824	0.112	nd			
Light											
*P*_n_ (O_2_)	(1,16)	28.611	**<0.001**	7.489	**0.015**	0.139	0.714	a	a	ab	b
*P*_n_ (CO_2_)	(1,16)	10.830	**0.005**	4.921	0.041	3.157	0.095	a	ab	b	b
*P*_g_ (O_2_)	(1,16)	17.108	**<0.001**	0.211	0.652	0.039	0.846	nd			
*P*_g_ (CO_2_)	(1,16)	7.219	0.017	1.141	0.301	0.792	0.387	nd			
*G* (CaCO_3_)	(1,16)	0.167	0.689	8.167	**0.011**	8.167	**0.011**	ab	ab	a	b
Chl *a*	(1,16)	1.009	0.330	0.646	0.433	0.091	0.767	nd			
**Winter**											
Dark											
*R*_d_ (O_2_)	(1,15)	0.257	0.619	18.389	**<0.001**	0.938	0.348	ab	a	b	a
*R*_d_ (CO_2_)	(1,15)	2.834	0.113	2.262	0.153	1.311	0.270	nd			
*G*_d_ (CaCO_3_)	(1,15)	0.017	0.898	3.794	0.070	0.676	0.424	nd			
Light											
*P*_n_ (O_2_)	(1,15)	62.325	**<0.001**	7.310	0.016	0.469	0.503	a	a	b	b
*P*_n_ (CO_2_)	(1,15)	48.847	**<0.001**	0.555	0.468	3.976	0.065	a	a	b	b
*P*_g_ (O_2_)	(1,15)	51.721	**<0.001**	1.056	0.321	0.000	1.000	a	a	b	b
*P*_g_ (CO_2_)	(1,15)	27.699	**<0.001**	5.228	0.037	1.082	0.315	ab	a	b	b
*G* (CaCO_3_)	(1,15)	0.344	0.566	12.396	**0.003**	0.344	0.566	nd			
Chl *a*	(1,15)	0.350	0.563	3.963	0.074	1.071	0.317	nd			
**Spring**											
Dark											
*R*_d_ (O_2_)	(1,15)	0.096	0.761	6.869	0.019	3.565	0.078	nd			
*R*_d_ (CO_2_)	(1,15)	0.126	0.728	3.142	0.097	0.005	0.944	nd			
*G*_d_ (CaCO_3_)	(1,15)	0.609	0.447	1.007	0.332	0.311	0.585	nd			
Light											
*P*_n_ (O_2_)	(1,15)	3.371	0.086	0.090	0.768	2.703	0.121	nd			
*P*_n_ (CO_2_)	(1,15)	0.143	0.711	0.548	0.470	17.780	**0.001**	a	b	ab	ab
*P*_g_ (O_2_)	(1,15)	4.235	0.057	0.448	0.514	4.661	0.047	nd			
*P*_g_ (CO_2_)	(1,15)	0.017	0.898	3.625	0.076	12.398	**0.003**	a	b	ab	ab
*G* (CaCO_3_)	(1,15)	1.734	0.208	0.444	0.515	7.864	0.013	nd			
Chl *a*	(1,15)	0.024	0.880	4.575	0.049	0.062	0.807	nd			

*R*_d_, dark respiration; *P*_n_, net production; *P*_g_, gross production; *G*_d_, net calcification in the dark; *G*, net calcification in the light. Bold type indicates Bonferroni-adjusted significance (*P* < 0.0125). Different letters (a and b) indicate significant difference between treatments: 400 *T*, 400 *T +* 3, 700 *T*, and 700 *T +* 3 (*P* < 0.0125, Tukey's HSD test); nd, no difference.

### Photosynthesis

#### Photosynthesis under ambient irradiance

Net (*P*_n_) and gross (*P*_g_) photosynthesis under ambient irradiance exhibited strong seasonal variations. *P*_n_ was highest in summer (0.38–0.66 μmol cm^−2^ h^−1^ in terms of O_2_ release or CO_2_ fixation), intermediate in autumn (0.14–0.33 μmol cm^−2^ h^−1^) and lowest in winter and spring (0.04–0.18 μmol cm^−2^ h^−1^). *P*_g_ also decreased from summer (0.67–0.93 μmol O_2_ or CO_2_ cm^−2^ h^−1^) to winter (0.14–0.25 μmol cm^−2^ h^−1^) and with intermediate values in autumn (0.34–0.43 μmol cm^−2^ h^−1^) and spring (0.18–0.29 μmol cm^−2^ h^−1^; Fig. 4).

*P*_n_ and *P*_g_ under ambient irradiance were strongly affected by pCO_2_ in summer, autumn and winter (Table 2), with a decline of 20–60% in *P*_n_ and 15–30% in *P*_g_ under elevated pCO_2_, relative to ambient pCO_2_. In spring, no effect of pCO_2_ or temperature alone was detected and only a significant interaction between pCO_2_ and temperature was observed in terms of CO_2_ fluxes. No effect of temperature was found on *P*_n_ and *P*_g_ whatever the season, except in summer on *P*_n_ in terms of CO_2_ fluxes.

The content of Chl *a* was not affected by pCO_2_ or temperature and did not show any significant difference among treatments (Table 2). It averaged 17.1 ± 0.8 μg Chl *a* cm^−2^ in summer, 17.0 ± 1.0 μg cm^−2^ in autumn, 18.0 ± 0.7 μg cm^−2^ in winter and 21.6 ± 1.1 μg cm^−2^ in spring.

#### Photosynthesis-irradiance curves

**Figure 5:**
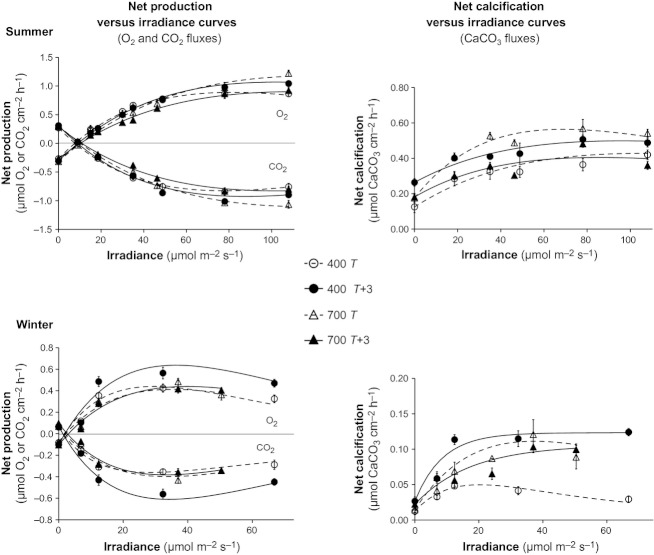
Net photosynthesis and calcification versus irradiance curves for *Lithophyllum cabiochae* in the four pCO_2_ and temperature treatments (400 *T*, 400 *T* + 3, 700 *T*, and 700 *T* + 3) in summer and winter. Net photosynthesis is expressed in terms of O_2_ production (negative values for respiration) and CO_2_ uptake (positive values for respiration). Data are means ± SE (*n* = 5, except in the 700 *T* + 3 treatment in winter, where *n* = 4).

The photosynthetic response of *L. cabiochae* to irradiance showed different patterns in summer and winter (Fig. 5). Maximum rate of gross photosynthesis (*P*_g_^max^) was about two to threefold higher in summer than in winter, while the initial slope (*α*) was lower in summer than in winter. The saturating (*E*_k_) and the compensation (*E*_c_) irradiances were about threefold and three to sixfold higher in summer than in winter, respectively. In winter, photoinhibition was observed with a decline of photosynthesis at irradiance levels higher than 40 μmol m^−2^ s^−1^.

In summer, the highest *P*_g_^max^ values were observed in the 700 *T* treatment with values *ca*. 130% higher than in the control both in terms of O_2_ and CO_2_ fluxes (Table 3). ANOVAs revealed significant interactions between pCO_2_ and temperature with contrasting responses according to the pCO_2_ levels. The parameters *α*, *E*_k_, and *E*_c_ were significantly affected by pCO_2_ with lower slopes (*α*) and higher irradiance values (*E*_k_ and *E*_c_) under elevated pCO_2_ relative to ambient pCO_2_.

**Table 3:** Comparison of *Lithophyllum cabiochae* net production and calcification versus irradiance curve parameters of the four treatments in summer and winter

					*P*-values from ANOVAs		
					
	400 *T*	400 *T* + 3	700 *T*	700 *T* + 3	pCO_2_	temperature	pCO_2_ × temperature
**Summer**							
O_2_							
*P*_g_^max^	1.16 ± 0.07^a^	1.35 ± 0.03^ab^	1.50 ± 0.04^b^	1.20 ± 0.07^a^	0.121	0.372	**<0.001**
*P*_n_^max^	0.90 ± 0.05^a^	1.05 ± 0.02^ab^	1.18 ± 0.04^b^	0.94 ± 0.08^a^	0.113	0.388	**0.001**
*α*	0.039 ± 0.002^a^	0.037 ± 0.002^ab^	0.034 ± 0.003^ab^	0.029 ± 0.001^b^	**0.002**	0.068	0.458
*E*_k_	30 ± 1^a^	37 ± 2^ab^	45 ± 3^b^	43 ± 3^b^	**<0.001**	0.384	0.063
*E*_c_	6.9 ± 0.5^a^	8.4 ± 0.6^ab^	9.6 ± 0.4^b^	9.3 ± 0.2^b^	**<0.001**	0.157	0.054
-CO_2_							
*P*_g_^max^	1.11 ± 0.08^a^	1.27 ± 0.04^ab^	1.40 ± 0.06^b^	1.12 ± 0.04^a^	0.212	0.331	**0.001**
*P*_n_^max^	0.84 ± 0.06^a^	0.96 ± 0.02^ab^	1.09 ± 0.07^b^	0.83 ± 0.03^a^	0.210	0.180	**0.001**
*α*	0.038 ± 0.002^a^	0.038 ± 0.002^a^	0.033 ± 0.002^ab^	0.030 ± 0.001^b^	**0.002**	0.336	0.445
*E*_k_	29 ± 1^a^	33 ± 1^ab^	43 ± 3^c^	38 ± 2^bc^	**<0.001**	0.960	**0.019**
*E*_c_	7.0 ± 0.4^a^	8.1 ± 0.3^ab^	9.3 ± 0.2^b^	9.8 ± 0.6^b^	**<0.001**	0.076	0.456
CaCO_3_							
*G*^max^	0.42 ± 0.02^ab^	0.50 ± 0.02^bc^	0.57 ± 0.02^c^	0.40 ± 0.01^a^	0.139	0.040	**<0.001**
*α*	0.008 ± 0.002^a^	0.007 ± 0.001^a^	0.015 ± 0.002^b^	0.007 ± 0.001^a^	**0.020**	**0.012**	0.040
*E*_k_	46 ± 9^nd^	37 ± 4^nd^	28 ± 3^nd^	33 ± 2^nd^	0.055	0.743	0.232
**Winter**							
O_2_							
*P*_g_^max^	0.52 ± 0.04^a^	0.71 ± 0.05^b^	0.49 ± 0.05^a^	0.54 ± 0.01^ab^	0.029	**0.012**	0.110
*P*_n_^max^	0.43 ± 0.04^ab^	0.61 ± 0.04^b^	0.41 ± 0.05^a^	0.43 ± 0.01^ab^	**0.021**	**0.025**	**0.068**
*α*	0.050 ± 0.002^ab^	0.056 ± 0.005^a^	0.038 ± 0.003^b^	0.037 ± 0.003^b^	**<0.001**	0.446	0.391
*E*_k_	10 ± 1^nd^	13 ± 1^nd^	13 ± 1^nd^	15 ± 1^nd^	0.038	0.038	0.607
*E*_c_	1.7 ± 0.1^a^	1.9 ± 0.2^a^	2.0 ± 0.1^a^	2.8 ± 0.2^b^	**<0.001**	**0.005**	0.074
-CO_2_							
*P*_g_^max¥^	0.43 ± 0.03^a^	0.64 ± 0.04^b^	0.44 ± 0.05^a^	0.48 ± 0.01^ab^	0.099	**0.006**	0.097
*P*_n_^max^	0.37 ± 0.02^a^	0.57 ± 0.04^b^	0.37 ± 0.05^a^	0.38 ± 0.01^a^	**0.023**	**0.012**	**0.020**
*α*	0.048 ± 0.004^nd^	0.056 ± 0.005^nd^	0.038 ± 0.005^nd^	0.040 ± 0.002^nd^	**0.009**	0.257	0.395
*E*_k_	9 ± 0^nd^	12 ± 1^nd^	12 ± 1^nd^	12 ± 1^nd^	0.041	**0.023**	0.135
*E*_c_	1.3 ± 0.2^a^	1.3 ± 0.2^a^	1.9 ± 0.2^ab^	2.5 ± 0.3^b^	**0.001**	0.191	0.191
CaCO_3_							
*G*^max¥^	0.05 ± 0.00^a^	0.12 ± 0.01^b^	0.10 ± 0.01^b^	0.11 ± 0.01^b^	**0.012**	**<0.001**	**<0.001**
*α*	0.005 ± 0.001^a^	0.011 ± 0.001^b^	0.007 ± 0.001^ab^	0.005 ± 0.001^a^	0.115	0.123	**0.002**
*E*_k_	8 ± 2^a^	9 ± 1^a^	13 ± 2^ab^	18 ± 2^b^	**<0.001**	0.079	0.228

*P*_g_^max^, *P*_n_^max^, and *G*^max^, maximal rate of gross production, net production and net calcification, respectively (μmol O_2_, C, and CaCO_3_ cm^−2^ h^−1^), *α*, initial slope of *P-E* or *G-E* curve (μmol cm^−2^ h^−1^ (μmol photons m^−2^s^−1^)^−1^), *E*_k_, light saturating point, and *E*_c_, compensation point (both in units of μmol photons m^−2^s^−1^).

Values are means ± SE (*n* = 5 except for the 700 *T +* 3 treatment in winter where *n* = 4). *P*-values from the two-way ANOVAs (df = 1,16 in summer and 1,15 in winter) are shown at right. Bold type indicates Bonferroni-adjusted significance (*P* < 0.025). Different subscripts (a, b, and c) indicate significant difference between treatments (*P* < 0.025, Tukey HSD post hoc tests); nd, no difference. Transformed data are indicated: ^¥^ log (*x*).

In winter, *P*_g_^max^ (both in terms of O_2_ and CO_2_ fluxes) was mainly affected by temperature, increasing with the 3°C rise in temperature under both ambient (140–150%) and elevated pCO_2_ (110%). *α* was significantly affected by pCO_2_ with lower slope under elevated pCO_2_ relative to ambient pCO_2_. No significant effect of pCO_2_ and temperature was found on *E*_k_, while *E*_c_ was mainly affected by pCO_2_, being higher under elevated pCO_2_.

### Calcification

#### Calcification under ambient irradiance

Net calcification in the dark (*G*_d_) exhibited strong seasonal changes with highest rates in summer (0.13–0.26 μmol CaCO_3_ cm^−2^ h^−1^), intermediate in autumn (0.03–0.05 μmol cm^−2^ h^−1^) and lowest in winter and spring (<0.03 μmol cm^−2^ h^−1^). In spring, *G*_d_ ranged between −0.01 and 0.00 μmol cm^−2^ h^−1^, with negative values corresponding to a net dissolution. ANOVAs revealed that *G*_d_ was significantly affected by temperature in summer but no significant difference among treatments was found at the other seasons (Table 2). Although non-significant (*P* = 0.07), an increase of *G*_d_ with temperature can be noted under both ambient and elevated pCO_2_ in winter.

#### Calcification-irradiance curves

The response of *L. cabiochae* calcification to irradiance is illustrated in Fig 5. The maximum rates of net calcification (*G*^max^) were four to eightfold higher in summer than in winter (Table 3). *α* was in the same order of magnitude at both seasons, while *E*_k_ decreased by a factor of 2–6 from summer to winter. Inhibition of calcification was evident in winter with a decline observed at irradiance >40 μmol m^−2^ s^−1^ under ambient temperature both in the 400 *T* and 700 *T* treatments.

*G*^max^ was significantly affected by the interaction between temperature and pCO_2_ in summer and mainly affected by temperature in winter (Table 3) with an increase in *G*^max^ with increasing temperature under ambient pCO_2_ and a decrease or no effect under elevated pCO_2_ (Table 3). *E*_k_ did not differ significantly among treatments in summer but was significantly affected by pCO_2_ in winter with higher values under elevated pCO_2_.

**Figure 6:**
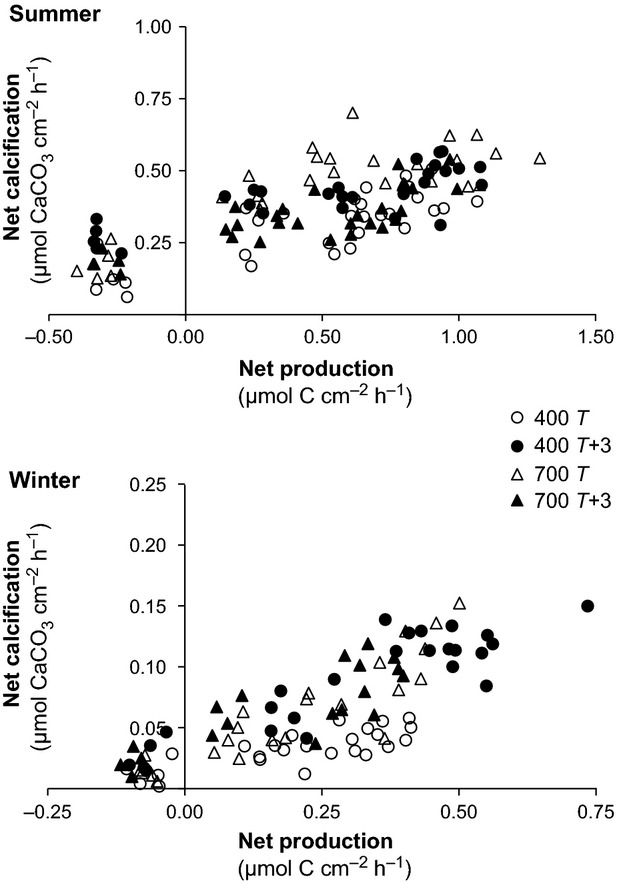
Relationships between net photosynthesis and calcification for *Lithophyllum cabiochae* in the four pCO_2_ and temperature treatments (400 *T*, 400 *T* + 3, 700 *T*, and 700 *T* + 3) in summer and winter.

Highly significant correlations were found between *G* and *P*_n_ in all treatments both in summer (*r* from 0.79 to 0.86, *P* < 0.001) and winter (*r* from 0.74 to 0.89, *P* < 0.001; Fig. 6).

## Discussion

### Response of respiration to elevated *p*CO_2_ and temperature

The changes in respiration rates observed in the present study were mainly related to temperature. The temperature dependence of respiration is well known for seaweeds (Lüning 1990) and has already been reported for several species of coralline algae with a trend of increasing respiration with increasing temperature (see Table 4 for a review). The seasonal changes in *R*_d_ in *L. cabiochae* are consistent with those previously reported for other species of temperate coralline algae. The threefold increase in *R*_d_ between winter and summer is comparable with that observed in the free-living coralline algae (maerl), *Lithothamnion corallioides* by Martin et al. (2006). *R*_d_ also responded positively to the 3°C rise in temperature during the colder seasons (autumn, winter, and spring) under both ambient and elevated pCO_2_ but with significant effect of temperature only observed in autumn and winter. Conversely, no or negative effects of increased temperature were observed in summer. Although non-significant, a decline of *R*_d_ with the 3°C rise in temperature occurred under elevated pCO_2_ (700 *T +* 3 treatment) in summer. It may be due to an increased sensitivity to high temperature (25°C) under elevated pCO_2_ leading to an early denaturation of enzymes at 25°C and a metabolic slowdown. This decline may also be the result of reduced photosynthesis in the 700 *T +* 3 treatment in summer and to the lower availability of photosynthates.

**Table 4:** Responses of coralline algal respiration, photosynthesis and calcification to elevated temperature (T) and *p*CO_2_, alone or in combination

Effects of:	↑ T	↑ pCO_2_	T×pCO_2_	References
On:				
**Respiration**				
Polar and sub-polar				
*Clathromorphum circumscriptum* (CCA)	↑			Adey (1973)
Temperate				
*Clathromorphum circumscriptum* (CCA)	↑			Digby (1977)
*Lithophyllum yessoense* (CCA)	↑			Ichiki *et al*. (2001)
*Lithothamnion coralioides* (R)	↑			Martin *et al*. (2006)
*Corallina officinalis (*ACA)		∩		Hofmann *et al*. (2012)
*Lithophyllum cabiochae* (CCA)	↑ or ―	―	― or ↓	Present study
Tropical and sub-tropical				
*Lithophyllum margaritae* (R)	↑			Steller *et al*. (2007)
*Hydrolithon* sp. (R)		―		Semesi *et al*. (2009)
**Photosynthesis**				
Polar and sub-polar				
*Clathromorphum circumscriptum* (CCA)	↑			Adey (1973)
Temperate				
*Clathromorphum circumscriptum* (CCA)	↑			Digby (1977)
*Corallina officinalis* (ACA)	↑			Digby (1977)
*Lithophyllum yessoense* (CCA)	↑			Ichiki et al. (2001)
*Phymatolithon calcareum* (R)	―			Wilson *et al*. (2004)
*Lithothamnion coralioides* (R)	↑			Martin *et al*. (2006)
*Corallina officinalis* (ACA)		― or ↓		Hofmann *et al*. (2012)
*Lithophyllum cabiochae* (CCA)	― or ↑	↓	―	Present study
Tropical and sub-tropical				
*Amphiroa foliacea* (ACA)		↑		Borowitska (1981)
*Amphiroa anceps* (ACA)		∩		Borowitska (1981)
*Lithophyllum margaritae* (R)	↑			Steller *et al*. (2007)
*Porolithon onkodes* (CCA)	↓	↓	↓↓	Anthony *et al*. (2008)
*Hydrolithon* sp. (R)		↑		Semesi *et al*. (2009)
*Corallina sessilis* (ACA)		↓		Gao & Zheng (2010)
**Calcification**				
Polar and sub-polar				
*Lithothamnion glaciale* (R)	↑	↓	―	Büdenbender *et al*. (1981)
Temperate				
*Bossiella orbiniana* (ACA)		∩		Smith & Roth (1979)
*Corallina pilulifera* (ACA)		↓		Gao *et al*. (1993)
*Lithothamnion coralioides* (R)	↑			Martin *et al*. (2006)
*Lithophyllum cabiochae* (CCA)	↑	―	↓	Martin & Gattuso (2009)
*Corallina officinalis* (ACA)^¥^		↓		Hofmann *et al*. (2012)
*Lithophyllum cabiochae* (CCA)	↑ or ―	↑ or ―	↓ or ―	Present study
Tropical and sub-tropical				
*Amphiroa foliacea* (ACA)		―		Borowitska (1981)
*Porolithon gardineri* (CCA)^ξ^	∩	↓		Agegian (1985)
*Lithophyllum margaritae* (R)	↑			Steller *et al*. (2007)
*Porolithon onkodes* (CCA)	―	↓	↓↓	Anthony *et al*. (2008)
*Lithophyllum* cf*. pallescens, Hydrolithon* sp. and*, Porolithon* sp. (R)^ϕ^		↓		Jokiel *et al*. (2008)
*Hydrolithon* sp. (R)		↓		Semesi *et al*. (2009)
*Neogoniolithon* sp. (CCA)		∩		Ries *et al*. (2009)
*Corallina sessilis* (ACA)		↓		Gao & Zheng (2010)
*Hydrolithon onkodes* (CCA)	―	↓	―	Johnson & Carpenter (2012)

Crustose CA (CCA), articulated CA (ACA), rhodoliths (R); ↑, increase; ↓ decrease; ↓↓ more-pronounced decrease; ―, no effect; ∩, parabolic response.

Respiration and photosynthesis rates presented here were determined from measurements of oxygen and *C*_T_ exchanges or fluorescence. Calcification rates were determined from alkalinity anomaly or buoyant weight techniques. Exceptions are indicated: ¥ growth determined from variations in fresh weight, ^ξ^ growth determined from red alizarin staining.

ϕ Rhodoliths used in this experiment consisted of a mixed CCA community including *Lithophyllum* cf*. pallescens, Hydrolithon* sp. and *Porolithon* sp.

No significant effect of pCO_2_ was detected on *L. cabiochae* respiration rates. This confirms recent findings that show no effect of pCO_2_ on respiration in various species of soft macroalgae (Zou et al. 2011) and in crustose coralline algae (Semesi et al. 2009). Although little is known on the response of algal respiration to increased CO_2_ concentrations, Zou et al. (2011) reported two possible concurrent responses: (1) the stimulation of respiration by an increase in respiratory substrates such as soluble carbohydrates due to enhanced photosynthesis and (2) a reduction in maintenance respiration due to a decrease in tissue nitrogen content (such as soluble protein and chlorophyll). However, in the present study, we found a decrease in photosynthesis with increasing pCO_2_ and no change in chlorophyll content.

### Response of photosynthesis to elevated *p*CO_2_ and temperature

The photosynthesis of *L. cabiochae* was significantly influenced by the season both in terms of production rates and photosynthetic characteristics. *P*_g_ under ambient irradiance was four to sixfold higher in summer than in winter while values of *P*_g_^ma*x*^ were two to threefold higher in summer than in winter. Such seasonal fluctuations are related to the changes of both temperature and irradiance which are fundamental parameters in the control of algal photosynthesis (Lüning 1990). The seasonal influence of temperature and irradiance on photosynthesis has been previously reported in coralline algal species such as *L. corallioides*, which exhibited values of *P*_g_^max^ twice higher in summer than in winter (Martin et al. 2006). Values of *E*_k_ and *E*_c_ for *L. cabiochae* were also considerably lower as the temperature and irradiance decreased from summer to winter. Conversely, the photosynthetic efficiency (*α*) was higher in winter than in summer suggesting a greater degree of shade acclimation in winter than in summer which is concordant with the decrease in irradiance levels between summer and winter.

In contrast with the seasonal influence of temperature on *L. cabiochae* photosynthesis, no effect of the 3°C warming was detected on *P*_g_ under ambient irradiance. However, significant and positive effects of temperature were found on *P*_g_^max^ in winter. This highlights the importance of examining the effect of temperature at various irradiances to assess the actual effect of warming on photosynthesis. A decline in photosynthesis can, however, occur at temperatures beyond the thermal optimum (Anthony et al. 2008). In summer, the 3°C warming effectively lead to reduced photosynthetic performance in *L. cabiochae* but only when combined to elevated pCO_2_. Interactive effects of pCO_2_ and temperature on photosynthesis have already been reported in the tropical crustose coralline alga *P. onkodes*, with an exacerbated drop in net productivity under elevated temperature and pCO_2_ (Anthony et al. 2008). In winter, under lower temperature levels, the 3°C rise in temperature was beneficial for photosynthesis under both ambient and elevated pCO_2_. However, the positive effect of warming on photosynthesis was more pronounced at ambient (*P*_g_^max^ increase of 140–150%) than at elevated pCO_2_ (110%), suggesting that the combination of elevated temperature and pCO_2_ may incur a physiological stress in winter or that algae did not recover their photosynthetic performance in this treatment yet.

The pCO_2_ exert a strong influence on the photosynthesis of *L. cabiochae*. Photosynthesis under ambient irradiance was negatively affected with a decline ranging from 15 to 30% for *P*_g_ and from 20 to 60% for *P*_n_ at elevated pCO_2_ in all seasons except in spring. Depressed photosynthesis caused by elevated pCO_2_ has already been reported in the articulated coralline alga *Corallina sessilis* (Gao et al. 1993; Gao and Zheng 2010). A decline in growth rate under elevated pCO_2_ has also been observed in several species of red algae (Israel et al. 1999; Israel and Hophy 2002). However, macroalgal species show mixed response to elevated pCO_2_. Enhancement of growth was found in the red alga *Porphyra yezoensis* at a pCO_2_ of 1000 μatm (Gao et al. 1991), while no response was reported in several species of Chlorophyta, Rhodophyta, and Phaeophyta (Israel et al. 1999; Israel and Hophy 2002). These authors attributed such non-responsiveness to the presence of CCMs which rely on HCO_3_^−^ uptake. The ability of macroalgae to use HCO_3_^−^ is related to its high availability in seawater relative to CO_2_. The enzyme carbonic anhydrase is involved in the CCM to convert the accumulated HCO_3_^−^ to CO_2_ for Rubisco, the enzyme that fixes CO_2_. Accordingly, the response of marine macroalgae to elevated pCO_2_ depends both on the extent to which HCO_3_^−^ is utilized. Some authors suggested that CO_2_ enrichment could still result in enhanced photosynthesis even for species that can effectively use HCO_3_^−^ because HCO_3_^−^ utilization requires energy (Gao et al. 1991). The decrease in photosynthesis at elevated pCO_2_ reported in the present study may be related to increased non-photochemical quenching and higher energy requirements under CO_2_ stress as suggested by Gao and Zheng (2010) for *C. sessilis*. Such decline in *L. cabiochae* photosynthesis does not appear to be related to concomitant response in pigment content as no change in Chl *a* concentration was observed under elevated pCO_2_. However, other key photosynthetic pigments are involved in red algae such as phycoerythrin and phycocyanin that may decrease with increasing pCO_2_ (Zou and Gao 2009). Interestingly, the response of photosynthesis to elevated pCO_2_ differs when algae are exposed to various irradiance levels or to ambient irradiance. The differential response in photosynthesis with increasing pCO_2_ under ambient irradiance (*P*_g_ and *P*_n_ at 400 μatm ≥ 700 μatm) and at saturated irradiance (*P*_g_^max^ and *P*_n_^max^ at 400 μatm ≤ 700 μatm) may result from the higher requirement of *C*_T_ for photosynthesis at higher irradiance levels, especially in summer. These differences may also be related to changes in photosynthetic energy conversion efficiency (*α*) with increasing pCO_2_. The significant decline in *α* under elevated pCO_2_ both in summer and winter may have slowed the metabolic process and reduce *P*_g_ and *P*_n_ under ambient irradiance, while it did not decrease the production capacity (*P*_g_^max^ and *P*_n_^max^) for the highest irradiance levels. Very few data are available on the effect of elevated pCO_2_ on the response of photosynthesis to irradiance but a recent study of Hofmann et al. (2012) reported a similar decline of *α* in response to increased pCO_2_ in the articulated coralline alga *Corallina officinalis*. In agreement with the decline in *α*, the *E*_k_ and *E*_c_ values of *L. cabiochae* were significantly increased under elevated pCO_2_ leading to higher values of the irradiance at which photosynthesis and respiration are compensated under elevated pCO_2_. This may have major implication for photosynthesis of algae growing in dim light environments. Accordingly, *L. cabiochae*, which mainly experiences irradiance levels close to the ambient culture irradiance (6–35 μmol m^−2^ s^−1^) is likely to be physiologically disadvantaged at future CO_2_ concentrations.

### Response of calcification to elevated *p*CO_2_ and temperature

Like photosynthesis, the process of calcification in *L. cabiochae* was related to irradiance. A strong relationship is found between irradiance and calcification (*G-E* curves). The *G-E* curves followed the same trend as the *P*-*E* curves reinforcing the hypothesis that calcification and photosynthesis processes are tightly linked (Pentecost 1978). Photosynthesis effectively affects calcification through the formation of the fibrous organic matrix of the cell walls that is needed for the deposition of calcite crystals in the cell wall of coralline algae and through changes in internal pH. Changes in pH that occur in the cell wall at the site of calcification are affected by both photosynthesis and respiration so that calcification is largely regulated by these metabolic activities (Smith and Roth 1979; Gao et al. 1993; Hurd et al. 2011). Increased (or decreased) pH due to photosynthesis (or respiration) leads to increased (or decreased) concentrations of CO_3_^2−^ and therefore can promote (or hinder) the precipitation of CaCO_3_ by increasing (or decreasing) the saturation state of CaCO_3_. Marked variations in both light and dark calcification rates of *L. cabiochae* were observed according to seasonal changes in temperature and irradiance, with maximal rates in summer, intermediate rates in autumn, and minimal rates in winter and spring. The low values observed in spring despite increased temperature and irradiance were attributed to the poor health condition at this period (Martin and Gattuso 2009). The environmental fluctuations in temperature and irradiance are known to exert a strong control on the rate of calcification of temperate coralline algae which decreases with decreasing temperature and irradiance from summer to winter as reported for *L. corallioides* (Potin et al. 1990; Martin et al. 2006). In the present study, the response of *L. cabiochae* calcification to elevated temperature and pCO_2_ differed among seasons, which shows that it is critical to take into consideration the interaction with seasonal changes of temperature and irradiance.

The 3°C rise in temperature was beneficial for *L. cabiochae* calcification when temperature were the lowest in autumn and winter, as already reported by Martin and Gattuso (2009) for diel net calcification when using the buoyant weight technique. At these seasons, a significant and positive effect of temperature alone was found on calcification under ambient irradiance. The calcification under ambient irradiance also increased with rising temperature in summer under ambient pCO_2_ although differences were non-significant. In contrast, under elevated pCO_2_, warming was detrimental to calcification due to a significant interaction between elevated pCO_2_ and temperature. Maximum rates of net calcification determined from the *G*-*E* curves in summer and winter also revealed a significant temperature effect as well as a significant interactive effect between temperature and pCO_2_ with an increase in *G*^max^ with increasing temperature under ambient pCO_2_ but a decrease (summer) or an absence of effect (winter) under elevated pCO_2_. Such response is similar to that observed in *P*_g_^max^ and *P*_n_^max^, confirming a close link between calcification and photosynthesis processes. Calcification in the dark was unaffected by increased temperature, except in summer where trend was close to that observed for light calcification with an increase in *G*_d_ with increasing temperature only under ambient pCO_2_. Interestingly, the detrimental effect of warming on calcification rates only occurred under high pCO_2_. Decreasing calcification in the 700 *T +* 3 treatment in summer is consistent with results of Martin and Gattuso (2009) who found a higher sensitivity of *L. cabiochae* to warming under elevated pCO_2_. A similar response was observed in the tropical crustose coralline alga *P. onkodes* with a positive effect of rising temperature (+3°C) under ambient pCO_2_ and a negative effect under elevated pCO_2_ (Anthony et al. 2008).

The response of calcification rates to elevated pCO_2_ differed according to the seasons but also to the light and dark conditions. In the dark, no effect of pCO_2_ alone was found on *G*_d_ at all seasons, while, in the light, the effect of pCO_2_ alone was observed on *G* only in summer. Interestingly, comparison between treatments among temperature levels (*T* or *T +* 3) revealed that *G* under elevated pCO_2_ were comparable or even higher (in summer) relative to *G* under ambient pCO_2_. In summer, *G* under ambient irradiance was 160% higher in the 700 *T* treatment than in the control. Higher values of *G*^max^ (140%) were also observed in the 700 *T* treatment relative to the control in summer. The initial slope (*α*) of the *G*-*E* curve was also higher in the 700 *T* treatment, suggesting a higher efficiency. Interestingly, the highest rate of diel net calcification measured by Martin and Gattuso (2009) between August 9 and September 8, 2006 was also observed in the 700 *T* treatment. Although most of studies reported negative effects of increased pCO_2_ on calcification of coralline algae (see Table 5; Gao et al. 1993; Anthony et al. 2008; Jokiel et al. 2008; Semesi et al. 2009; Gao and Zheng 2010; Büdenbender et al. 2011; Johnson and Carpenter 2012), some studies found no (Borowitzka 1981; Martin and Gattuso 2009) or positive effects at elevated pCO_2_ (Smith and Roth 1979; Ries et al. 2009). Ries et al. (2009) reported a twofold increase in net calcification at intermediate pCO_2_ levels (605 and 903 μatm) relative to the control in the tropical crustose coralline alga *Neogoniolithon* sp. while Smith and Roth (1979) reported higher rates of calcification at 1300 μatm than at lower and higher pCO_2_ in the articulated coralline alga *Bossiella orbigniana*. Such response may be related to the ability of the algae to maintain an elevated pH at the site of calcification despite reduced external pH which would facilitate CaCO_3_ precipitation. Higher calcification rates of *L. cabiochae* in the 700 *T* treatment in summer corresponds to higher rate of photosynthesis (*P*_g_^max^ and *P*_n_^max^). The mitigating role of photosynthesis on calcification is further confirmed by the lack of effect of elevated pCO_2_ on calcification in the dark. Changes in pH related to metabolic processes (photosynthesis or respiration) may occur in the cell walls at the site of calcification but also in the diffusion boundary layer between the algal surface and external seawater (Hurd et al. 2011). The ability of coralline algae to increase pH in their cell walls and at their surface via photosynthesis may thus increase their resilience to elevated pCO_2_. Coralline algae are also able to maintain calcification in the dark even at the relatively low pH values generated by respiration. Due to their exposure to a wide range of pH, coralline algae may have some physiological flexibility to acclimate to elevated pCO_2_. Following an earlier suggestion of Digby (1977), Hofmann et al. (2012) proposed that carbonic anhydrase may also play a role in the calcification of coralline algae by catalyzing the conversion of CO_2_ into HCO_3_^−^ and then CO_3_^2−^. The stimulation of carbonic anhydrase activity could therefore also help preventing a decrease in calcification at elevated pCO_2_. However, carbonic anhydrase is also used by photosynthesis to convert HCO_3_^−^ to CO_2_. This implies that processes of photosynthesis and calcification may thus be concurrent. The maintenance or enhancement of calcification rates under elevated pCO_2_ in *L. cabiochae* may thus be detrimental to photosynthesis, as indicated by reduced photosynthesis under elevated pCO_2_.

## Conclusion

Coralline algae are considered to be among the most vulnerable organisms to ocean acidification due to the high solubility of their high-Mg calcite skeleton. However, the present findings provide evidence of the ability of *L. cabiochae* to maintain or even enhance its rate of calcification under increased pCO_2_. The metabolic cost of maintaining calcification may still be detrimental to other physiological processes such as photosynthesis. We also demonstrated a particular interaction between ocean acidification and warming leading to decreased physiological rates especially during the warmer season at temperature beyond the thermal optimum. These data provide insights into the potential for physiological acclimation to future environmental changes in coralline algae. However, the present study reports only on the physiological response of healthy specimens to ocean warming and acidification, while these environmental changes may affect the vulnerability of coralline algae to other stresses such as pathogens and necroses that can cause major dissolution (Martin and Gattuso 2009), which would have major consequence for the ability of *L. cabiochae* population to precipitate calcium carbonate. Given the critical ecological functions of coralline algae in the coralligenous habitat, future conditions of pCO_2_ and temperature in the next decades and century ahead may have major consequences for the sustainability of Mediterranean coralligenous habitats and critical ecological implications for coastal Mediterranean ecosystems.
